# The pattern of emergency department length of stay in Saudi Arabia: an epidemiological Nationwide analyses of secondary surveillance data

**DOI:** 10.3389/fpubh.2023.1265707

**Published:** 2023-12-12

**Authors:** Abdullah A. Alharbi, Mona Muhayya, Reem Alkhudairy, Ahmed A. Alhussain, Mohammed A. Muaddi, Ahmad Y. Alqassim, Reem S. AlOmar, Mohammed K. Alabdulaali

**Affiliations:** ^1^Family and Community Medicine Department, Faculty of Medicine, Jazan University, Jazan, Saudi Arabia; ^2^Ministry of Health, Riyadh, Saudi Arabia; ^3^Department of Family and Community Medicine, College of Medicine, Imam Abdulrahman Bin Faisal University, Dammam, Saudi Arabia

**Keywords:** emergency department, length of stay, key performance indicator, epidemiology, public health, Saudi Arabia

## Abstract

**Background:**

Emergency department length of stay is a vital performance indicator for quality and efficiency in healthcare. This research aimed to evaluate the length of stay patterns in emergency departments across Saudi Arabia and to identify predictors for extended stays. The study used secondary data from the Ministry of Health’s Ada’a program.

**Methods:**

Using a retrospective approach, the study examined data from the Ada’a program on emergency department length of stay from September 2019 to December 2021. These data covered 1,572,296 emergency department visits from all regions of Saudi Arabia. Variables analyzed included quality indicators, year of visit, shift time, hospital type, and data entry method. The analysis was conducted using multiple linear regression.

**Results:**

The study found that the median length of stay was 61 min, with significant differences among related predictors. All associations were significant with a value of p of less than 0.001. Compared to 2019, the length of stay was notably shorter by 28.5% in 2020 and by 44.2% in 2021. Evening and night shifts had a shorter length of stay by 5.9 and 7.8%, respectively, compared to the morning shift. Length of stay was lower in winter, summer, and fall compared to spring. Patients in levels I and II of the Canadian Triage and Acuity Scales had longer stays than those in level III, with those in level I reaching an increase of 20.5% in length of stay. Clustered hospitals had a longer length of stay compared to the non-clustered ones. Pediatric hospitals had a 15.3% shorter stay compared to general hospitals. Hospitals with data entered automatically had a 14.0% longer length of stay than those entered manually. Patients admitted to the hospital had a considerably longer length of stay, which was 54.7% longer compared to non-admitted patients. Deceased patients had a 20.5% longer length of stay than patients discharged alive.

**Conclusion:**

Data at the national level identified several predictors of prolonged emergency department length of stay in Saudi Arabia, including shift time, season, severity level, and hospital type. These results underline the necessity of continuous monitoring and improvement efforts in emergency departments, in line with policy initiatives aiming to enhance patient outcomes in Saudi Arabia.

## Introduction

A key performance indicator (KPI) for emergency department (ED) visits is the length of stay (LOS; measured in minutes) from admission to the ED until the final disposition of the visit (1). Although measuring quality in EDs presents unique challenges, numerous studies suggest that the LOS is one of the central predictors for measuring outcomes (2–4). The importance of this indicator is that previous studies have shown that the LOS for a patient in the ED is associated with the outcome of the case, including mortality and hospital admission rates (5). In addition, there is a close inverse relationship between service satisfaction and LOS in the ED (6). Many factors have been found to affect the ED LOS, including the severity of patients’ illnesses, patients’ flow, the number of advanced tests performed in the ED, race, day, and time of the visit to the ED (7–12). Additionally, there are many consequences of prolonged LOS at ED visits, such as increased morbidity and mortality, increased costs, delays in admission for treatment, poor patient outcomes, and delays in treating additional patients (2, 13–15). However, to assess the quality and efficiency of the ED and to propose solutions for prolonged LOS, it is essential to obtain baseline data regarding the current LOS during ED visits and the factors that influence it in Saudi Arabia.

Internationally, efforts have been made in some countries to measure and benchmark the LOS in healthcare settings. For instance, a study conducted in Germany at the national level reported a median LOS of 171.3 min (1). Similarly, in the United States, the median LOS was reported to be 146 min (15). However, in Saudi Arabia, research in this area has been limited to single institutions and has not been based on routinely collected secondary data gathered for surveillance and performance measurement purposes (16). Therefore, there is a need for comprehensive and systematic studies that utilize standardized data collection methods to assess and benchmark LOS in different healthcare settings in Saudi Arabia, in line with international practices. Such studies can provide valuable insights for policymakers and healthcare providers to improve patient care, optimize resource utilization, and enhance overall healthcare system performance.

The National Center for Performance Management, known as the Ada’a program, was established by the Ministry of Health in 2017 and serves as a comprehensive tool to measure, improve, and monitor the current healthcare system in Saudi Arabia (17). Hospitals are gradually adopting the Ada’a program, and a dedicated database has been developed to collect data on its implementation (18). One of the KPIs of the Ada’a program is the LOS in various healthcare settings, which is closely monitored for performance measurement (19–21). The current Saudi healthcare system is unsustainable due to the rising demand for services, an economic downturn, and an increasingly older adult population. Therefore, Saudi Arabia is launching a new healthcare transformation. The new model of care, under a new healthcare transformation, is a part of the Saudi Vision 2030 initiative, which aims to privatize the healthcare system to enhance its quality and value through the introduction of national insurance coverage to all citizens, whereby the system’s financial administration remains under the oversight of the Ministry of Health. Hence, the new model of care is a hybrid model including private and governmental sectors (22). Ada’a is a program designed to implement the new healthcare of Vision 2030 in an organized fashion, with the goal of minimizing disruption to the overall system. Therefore, this is the first study to examine the pattern of ED LOS across all 13 administrative regions of Saudi Arabia, utilizing secondary data from the Ada’a program with a sample of over 1 million ED visits. The study also aims to estimate the predictors affecting LOS in these national data.

## Methods

### Data source, design, and participants

The data for this study were obtained from the Ada’a Health Program, a comprehensive nationwide data collection initiative that gathers secondary data from hospitals across Saudi Arabia. Patient information was typically recorded manually or electronically, and participating hospitals submitted special data forms using a unified barcode to the Ada’a database. The study included all variables with a total of 1,572,296 patients. These patients had visited EDs in 110 hospitals across all 13 administrative regions of Saudi Arabia. The sample was restricted to patients who were triaged and classified based on disease severity using the widely accepted Canadian Triage and Acuity Scale, as defined by the Ministry of Health protocols, and were enrolled in the Ada’a Health Program from September 2019 to December 2021. This ensured a comprehensive representation of ED visits during the study period, making the findings of this study robust and generalizable to the broader population of ED patients in Saudi Arabia.

### Ethical considerations

This study was reviewed by the Ministry of Health institutional review board (IRB 22–54 E) and Imam Abdulrahman Bin Faisal University’s institutional review board (IRB-2023-01-304) and was conducted by utilizing secondary aggregated data with no identifying or personal information.

### Measurements

The study used one of the KPIs described by the Ada’a Health Program for each patient, namely, “door to disposition,” which we define as the total time (in minutes) a patient spent from registration until a care decision was determined (i.e., discharge, admission, or transfer). The analysis included the entire dataset collected by the Ada’a Health Program, and the dataset included the shift during which the visit occurred: morning shift (8:00–16:00), evening shift (16:00–00:00), and night shift (00:00–8:00); the season of the visit: winter (December, January, and February), spring (March, April, and May), summer (June, July, and August), and autumn (September, October, and November); the severity of the visit based on the Canadian Triage and Acuity Scale (level I resuscitation, level II emergent, level III urgent, level IV less urgent, and level V non-urgent); the type of hospital (general, maternity, pediatric, and trauma center); discharge outcomes [home, transfer to other hospitals, discharge against medical advice (DAMA), and leave against medical advice (LAMA)]; the method of data entry (manual or automated data registry); the admission type [ward or intensive care unit (ICU)]; the hospital status regional health departments (i.e., hospital is not clustered, or hospital is clustered under new the healthcare transformation); and, finally, if patients were admitted to hospital or died during admission.

### Statistical analysis

Descriptive statistics were used to analyze the data. Due to its highly positive skewness, the outcome variable, LOS, was expressed as medians and interquartile ranges. Bivariate associations were performed through a series of non-parametric tests, which included both the Mann–Whitney *U* tests (where the categorical variable was binary) and the Kruskal–Wallis tests to derive levels of significance showing differences in the median LOS. To assess the performance of each indicator on the LOS outcome variable, a linear regression model was used after the log transformation of the outcome was performed. Statistical significance was set at a value of *p* of 0.05, and Stata statistical software version 16 (StataCorp, College Station, TX, United States) was used.

## Results

Table 1 presents the patterns and characteristics of ED visits. The median LOS in the ED was 61 min, and the interquartile range was 30–126 min, whereas the mean and standard deviation were 103 ± 130 min. Of the 1,572,296 total visits, 66.0% occurred in 2021, 25.1% in 2020, and 8.9% in 2019 (2019 only included data from September to December). The ED visit distribution related to shifts was similar, with 38.0% in the evenings, 33.0% in the mornings, and almost 29.0% at night. Most ED visits occurred during the spring season, whereas only 7.6% occurred during the fall. Concerning case severity, most were at level III, followed by level IV (46.7 and 35.1%, respectively), while <1.0% were at level I. Hospitals that belonged to a cluster formed 45.6% of the data. Approximately 71.0% of ED visits occurred in general hospitals, whereas only 6.0% occurred in pediatric hospitals. Although data were entered using manual and automated methods, nearly 91.0% were manually entered. Admission type for those admitted to the hospital was provided for a subset of 226,928 ED visits, revealing that nearly 92.0% were admitted to general wards and 8.0% were admitted to the ICU. Discharge outcome was provided for 1,344,848 participants, revealing that 94.0% were discharged to home, approximately 1.0% were transferred to another hospital, approximately 2.0% were discharged against medical advice (DAMA), approximately 3.0% left against medical advice (LAMA), and 0.2% died while in the ED.

**Table 1:** Patterns and characteristics of ED visits.

Characteristics of ED visit	Total number/frequency of visit, N = 1,572,296	Percentage SD IQR
Year		
2019	139,821	8.89
2020	394,752	25.11
2021	1,037,723	66.00
Time of shift		
Morning shift	520,697	33.12
Evening shift	597,823	38.02
Night shift	453,776	28.86
Seasons		
Winter	186,145	11.84
Spring	1,113,553	70.82
Summer	158,590	10.09
Fall	114,008	7.25
Severity of visit based on the Canadian triage and acuity scale		
Resuscitation (level I)	11,182	0.71
Emergent (level II)	64,957	4.13
Urgent (level III)	733,736	46.67
Less urgent (level IV)	552,170	35.12
Non-urgent (level V)	210,251	13.37
Clusters		
Non-clustered	860,897	54.75
Clustered	711,399	45.25
Type of hospital or department		
General hospital	1,112,398	70.75
Maternity hospital	369,187	23.48
Pediatric hospital or department	90,711	5.77
Data entry		
Manual data registry	1,426,296	90.71
Automated data registry	146,000	9.29
Admission type		
ICU^#^	18,746	8.26
Ward	208,181	91.74
Discharge outcomes		
Home	1,264,174	94.00
Transfer to another hospital	14,177	1.05
LAMA^&^	23,554	1.75
DAMA^*^	40,120	2.98
Deceased	2,823	0.21
KPI (door to disposition, minutes)		
Admitted to hospital		
No	1,345,369	85.57
Yes	226,927	14.43
Mortality		
No	1,569,473	99.82
Yes	2,823	0.18

IQR: interquartile range. ^#^ICU: intensive care unit. ^&^LAMA: leave against medical advice. *DAMA: discharge against medical advice.

Table 2 presents the median minutes and interquartile ranges of the ED visits. The median LOS had a statistically significant decrease from 2019 to 2021 (*p* < 0.001; from 82 min in 2020 to 56.58 min in 2021); the trend for value of *p* was also significant at <0.001. Compared with the morning shift, the median LOS was significantly shorter for the evening and night shifts. Spring was seen to have the lowest median LOS. Compared to patients in level III of the Canadian Triage and Acuity Scale, the median LOS was significantly longer for level I patients, followed by level II patients, and the lowest median LOS was for level V patients. The results also showed that the median LOS was significantly longer for hospitals that were part of regional clusters. With regard to the type of hospital, the lowest median LOS was found to be in pediatric hospitals. The utilization of an automated data registry tended to show a more prolonged median LOS. Compared with participants admitted to the general wards, the median LOS was significantly longer for those admitted to the ICU. Examination of discharge outcomes showed that the median LOS was highest for patients who had LAMA according to their medical records, followed by patients who died. Similarly, patients who had been admitted to hospitals from the ED consequently had a much longer median LOS (130 min) compared to those who were not (55 min). All associations were significant at the *p* < 0.001 level.

**Table 2:** LOS median minutes by characteristic for ED visits in Saudi Arabia.

Characteristics of ED visit	Median (minutes)	IQR (minutes)	*p* value
ED length of stay	61.00	(30–126)	
Year			
2019	82.00	(40–158)	<0.001
2020	71.00	(34–140)	
2021	56.58	(28–115)	
Time of shift			
Morning shift	66.00	(32–137)	<0.001
Evening shift	60.00	(30–121)	
Night shift	59.00	(30–120)	
Seasons			
Winter	65.00	(29–132)	<0.001
Spring	60.00	(30–124)	
Summer	63.00	(32–130)	
Fall	66.00	(32–133)	
Severity of visit based on the Canadian triage and acuity scale			
Resuscitation (level I)	142.00	(59–277)	<0.001
Emergent (level II)	117.00	(50–232)	
Urgent (level III)	80.00	(39–158)	
Less urgent (level IV)	50.00	(26–96)	
Non-urgent (level V)	41.00	(21–71)	
Cluster status			
Non-clustered	60.00	(30–121)	<0.001
Clustered	64.00	(30–133)	
Type of hospital or department			
General hospital	63.00	(30–134)	<0.001
Maternity hospital	59.00	(32–108)	
Pediatric hospital	54.00	(27–110)	
Data entry			
Manual data registry	60.00	(30–123)	<0.001
Automated data registry	75.00	(39–153)	
Admission type: 226928			
ICU^#^	149.00	(72–273)	<0.001
Ward	128.00	(61–243)	
Discharge outcomes: 1344848			
Home	54.00	(28–106)	<0.001
Transfer to another hospital	59.00	(23–190)	
LAMA^&^	97.00	(50–166)	
DAMA^*^	84.00	(44–155)	
Deceased	95.00	(50–172)	
Admitted to hospitals			
No	55.00	(28–109)	<0.001
Yes	130.00	(62–245)	
Died during the visits			
No	61.00	(30–126)	<0.001
Yes	95.00	(50–172)	

N = 1,572,296. IQR: interquartile range. ^#^ICU: intensive care unit. ^&^LAMA: leave against medical advice. *DAMA: discharge against medical advice.

**Figure 1:**
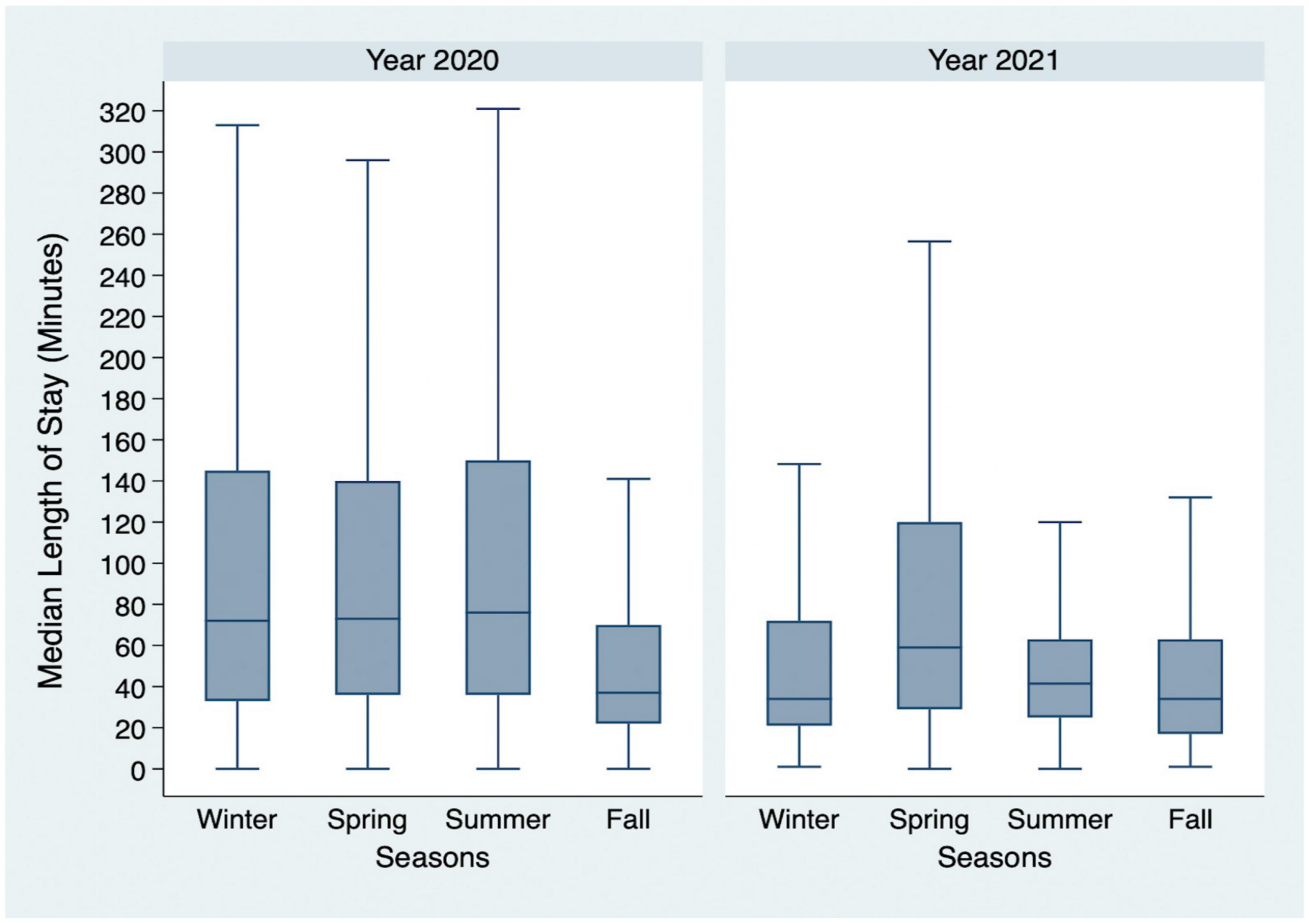
Seasonal variations of the median length of stay for emergency department visits according to years. *The year 2019 is not shown, as our study included data from September to December for this year.

Figure 1 depicts the findings of our study regarding the seasonal trends in the median LOS for ED visits over different years. We observed that the median LOS varied across seasons, with notable fluctuations observed between years.

The results of the multiple linear regression analysis of the factors associated with the estimated log-transformed LOS in minutes are shown in Table 3. Compared to 2019, LOS during ED visits in 2020 and 2021 was significantly shorter by 28.5 and 44.2%, respectively (*p* < 0.001). Compared to the morning shift, the ED LOS for the evening and night shifts was significantly shorter by 6.0 and 9.0%, respectively (*p* < 0.001). The ED LOS in the winter, summer, and fall seasons was significantly shorter than in the spring season by 22.0, 11.0, and 34.0%, respectively (*p* < 0.001). Compared to severity level III, the ED LOS for severity levels I and II was significantly longer by 21.0 and 11.0%, respectively. In comparison, severity levels IV and V were significantly shorter by 32.0 and 57.0%, respectively (*p* < 0.001). The ED LOS for hospitals in the cluster group was 6.0% shorter than those in the regional health department group (*p* < 0.001). The LOS for maternity and pediatric hospitals was significantly shorter, by 5.0 and 15.0%, respectively, compared to general hospitals (*p* < 0.001). Compared with ED visit data that was entered by manual methods, the LOS data entered by automated methods was significantly longer by 14.0% (*p* < 0.001). Compared with participants who were discharged from the ED, the ED LOS for those who were admitted to the hospital was significantly longer by 55.0%. Compared with participants who survived, the ED LOS for those who died in the ED was significantly longer by 20.0%.

**Table 3:** Multiple linear regression analysis of the factors associated with the length of stay.

log_LOS	Coefficient	Standard error	*t*-value	[95% confidence interval]	*P* value
: Base 2019b	0	.	.	.	
2020	−0.285	0.004	−74.37	(−0.293, −0.278)	<0.001
2021	−0.442	0.004	−108.14	(−0.45, −0.434)	<0.001
: base morning shift	0	.	.	.	
Evening shift	−0.059	0.002	−32.59	(−0.062, −0.055)	<0.001
Night shift	−0.078	0.002	−40.62	(−0.082, −0.074)	<0.001
: base spring	0	.	.	.	
Winter	−0.219	0.003	−75.31	(−0.225, −0.214)	<0.001
Summer	−0.11	0.003	−38.36	(−0.116, −0.105)	<0.001
Fall	−0.343	0.004	−82.96	(−0.351, −0.334)	<0.001
: Base III	0	.	.	.	
I	0.205	0.009	21.82	(0.187, 0.224)	<0.001
II	0.109	0.004	27.55	(0.101, 0.117)	<0.001
IV	−0.322	0.002	−184.35	(−0.325, −0.318)	<0.001
V	−0.57	0.002	−237.36	(−0.575, −0.566)	<0.001
: Base regional health department	0	.	.	.	
Cluster	0.064	0.002	40.99	(0.061, 0.067)	<0.001
: Base general hospital	0	.	.	.	
Maternity hospital	−0.048	0.002	−26.14	(−0.052, −0.044)	<0.001
Pediatric hospital	−0.153	0.003	−45.56	(−0.159, −0.146)	<0.001
: Base manual	0	.	.	.	
Automated	0.143	0.003	52.53	(0.137, 0.148)	<0.001
Admitted to hospital	0.547	0.002	231.77	(0.543, 0.552)	<0.001
Deceased	0.205	0.019	10.98	(0.168, 0.242)	<0.001
Constant	4.679	0.004	1045.28	(4.67, 4.688)	<0.001

## Discussion

The purpose of this study was to assess median ED LOS and examine related factors in Saudi Arabia using national-level data from the Ministry of Health’s Ada’a Program. We identified patterns of LOS and factors predicting prolonged LOS, including shift, season, severity level, and type of hospital. Our study, the first to assess LOS in Saudi Arabia using data of this magnitude, observed a lower median LOS compared to other studies from different countries (1, 15). A prolonged LOS in the ED may have several adverse consequences, for example, at the system level, it may increase healthcare spending and admissions to hospitals, while at the patient level, it may impact patient safety and outcomes, such as increased mortality rates (5). The median LOS of 61 min observed from these data was lower than that observed in other international studies utilizing nationwide data. For example, in Germany, the median LOS was 171.3 min, while in the United States, it was 146 min (interquartile range = 84–242) (1, 15). Differences in healthcare systems, patient populations, ED protocols, and contextual factors may have contributed to these findings (23). Further research in diverse populations and settings would enhance our understanding of ED LOS and its implications for patient care (3). It is worth noting that inherent differences in data collection methods and the timing of data collection, such as the US study being based on data collected prior to the pandemic era, may have played a role in these differences (1, 15).

This study observed a clear decreasing trend in LOS over time (Figure 1). The continuous monitoring provided by the Ada’a program enhances the improvement and development of policies and practices and ensures fast and effective responses to patients. The data presented may reflect the success of this initiative and document a reduced LOS over time. However, the impact of COVID-19 cannot be underestimated. The measures applied by the Saudi government in terms of nationwide restrictions beginning in March 2020, accompanied by the pressure to minimize infection either by tending to patients rapidly and effectively or by referring patients belonging to levels IV and V of the Canadian Triage and Acuity Scale to primary care centers whenever possible, could have contributed to the decreased LOS. This rapid response by the Saudi government resulted in one of the lowest COVID-19 infection and mortality rates globally and positively affected overall healthcare services, as demonstrated by a Saudi study (22, 24). This finding mimics the results of a population-based Canadian study that focused on inpatient LOS (25). For studies of ED visits alone across years, our findings are contradictory either due to those studies being on single institutions, the inclusion of specific illnesses, or the inclusion of only very severe cases (26–28).

In our study, we found that the evening and night shifts in EDs had shorter LOS compared to the morning shifts. This is consistent with previous research indicating that patients who visit in the morning shifts, which often include rotating medical students, may experience prolonged patient care. Different studies have shown that the presence of medical students in the ED can affect the LOS, particularly in hospitals with residents and medical students (29, 30). Additionally, the morning shift tends to include patients with a lower triage level, either because of trauma or severe illness. Although this was not examined in the present study, previous studies from Saudi Arabia reported higher rates of admissions during the morning (31). As one department of many within a hospital, shift differentials are bound to exist due to inpatient beds being in greater demand during daytime hours, according to a study that measured this aspect (32). The ED LOS may be affected by the standard protocol for inpatients to be discharged after morning rounds and procedures; therefore, there is a delay in available beds. Additionally, the workload varies by shift, with morning ED staff covering other departments and procedure areas as well as the ED. In addition, evening and night shift staff may be on call and dedicated to the ED alone when inpatient beds are more available, resulting in patients being admitted to wards sooner.

Interestingly, upon examination of seasonality, the LOS pattern shows that, for 2020, a spike in LOS during the summer was observed, while in 2021, the spike was in the spring. This could be attributed to the holy months of Ramadan and Eid, where the shift turnover is more rapid than that of the other months; hence, the change between physicians is more frequent. With frequent changes, improper handovers may occur, delaying care delivery and increasing waiting hours. However, the literature on seasonality shows differing results in terms of the number of visits and LOS, although the findings were attributed to individual patient clinical characteristics such as age and complaint, which could not be ascertained given the current data. Furthermore, the data and findings were obtained before the pandemic (31–33).

Concerning Canadian Triage and Acuity Scale severity levels, we found that those categorized at levels I and II had a longer LOS in the ED than level III patients, whereas for patients in levels IV and V, the LOS was much lower. These results are consistent with the Canadian Triage and Acuity Scale objectives. Traumatic and emergent cases would require extensive treatment and diagnostic and imaging requests to cater to their critical condition. Similar findings have been reported in Saudi Arabia and have attributed the lower LOS in the non-urgent levels to the high number of patients leaving unnoticed by medical staff (34). Overcrowding and overuse of EDs by patients with non-urgent complaints that are more suitable to be seen in a primary care setting have long been declared an issue among ED physicians in Saudi Arabia. The Ministry of Health has already put in place new initiatives to tackle this problem, such as the new “urgent care” model, which aims to streamline the pathway of health services. This, in turn, should alleviate the burden of EDs and improve patient outcomes (35).

Although an increased LOS is not necessarily an indicator of lower quality of care, it is an important factor leading to ED overcrowding (36, 37). The Ministry of Health’s transformation strategy aims to improve healthcare efficiency and avoid overcrowding through several approaches, such as creating clusters of providers currently being implemented in several phases (38). Health clusters provide comprehensive healthcare services through an integrated network of healthcare professionals operating under a single administrative framework that includes curative and preventive components. A cluster is comprised of many levels of healthcare services, including primary care, hospitals, and specialized facilities, in order to facilitate access to any necessary services (39). Clustering has been proposed to improve capacity, financing, governance, and delivery of care under the Kingdom’s “Vision 2030” strategic plan objectives for health (38, 40). However, clustering is still in a transitional phase and is only being implemented in selected healthcare centers. This could explain the longer LOS in clustered hospitals that are part of the initial phase, as there may be temporary challenges that lead to delays and disruptions in the flow of patients. Additionally, healthcare providers at these centers may need to spend more time coordinating care and adjusting to the new system, which can also contribute to longer LOS.

Another important finding in examining variables related to LOS was the type of health facility. A longer LOS was observed in EDs at general hospitals compared to maternity and pediatric hospitals. This finding is comparable with that of another study examining LOS in general versus specialty hospitals (41). General hospital EDs tend to deal with highly diverse types of patients and health conditions, some of which require longer time, such as road traffic injuries, whereas specialty hospitals tend to have specialists dealing with specific types of patients and a limited number of health conditions (42). The higher efficiency of medical and paramedical personnel can be attributed to the routinization and repetition of healthcare practices, which are more likely to be obtained in specialized centers than in general ones. Contradictory findings have been documented in another study conducted in Boston, US, which found a longer LOS in pediatric EDs than in general EDs for pediatric patients (43). This was explained by the higher number of pediatric medical conditions observed in pediatric EDs than in general EDs. In Saudi Arabia, few studies have been conducted to examine LOS (44, 45); however, cross-country comparisons should be made with caution because of the large differences in terms of cultural and behavioral factors, and most importantly, differences in healthcare systems.

Data registry/entry type was another important influencing factor for ED LOS, in which longer LOS was observed in hospitals using automated data registry/entry type than those entering data manually. In addition to the questionable accuracy of the data entered manually compared to those entered automatically, automated data registry/entry is usually practiced in advanced hospitals where more specialized lab tests and imaging are provided. In addition, using advanced services and techniques in these centers might prolong the LOS (46, 47). Another possible explanation is that advanced hospitals are more likely to be attractive training centers for students and newly graduating medical and paramedical professionals, which several studies have identified as factors leading to increased LOS (29, 48).

Our data found that patients who were either DAMA or LAMA had a median LOS that was longer than those who were discharged to home, in which the median time for patients to decide to leave the ED was approximately 90 min. Although this study did not explore the reasons for DAMA and LAMA, it may be that the long wait time was one of the reasons for leaving the ED untreated, as evidenced in the previously published literature (49). This patient population is worthy of further investigation because it affects hospital resource utilization as this population is more likely to re-visit the ED for the same complaint.

Regarding policy recommendations, we suggest that this study provides important insights for policymakers. First, there is a need for continued expansion of primary care, especially when co-located with EDs. This is important if we consider that 48% of ED visits are classified as either less urgent or less urgent on a triage scale. Second, adopting practices that studies have proven to help the patient flow from door to disposition time should be encouraged. These concepts include, but are not limited to, point-of-care and medical team evaluation (50, 51). Third, health facility administrators should be encouraged to apply accredited standards, such as those found in organizations such as the Joint Commission on Accreditation of Healthcare Organizations, in measuring the effectiveness and efficiency of the ED. Fourth, the seasonal variation we found in this study emphasizes the call for a backup policy in EDs at the national level to ensure adequate staffing to provide sufficient care in a timely manner (52). Fifth, management of workflow, especially among shifts, is crucial to overcome prolonged LOS; therefore, administration at appropriate levels should develop and enforce proper handover protocols to mitigate the effects of ED crowding on the observed quality of care and outcomes (53). Finally, hospital leadership and medical practitioners must establish methods to measure, analyze, and address identified quantitatively and recurrent causes of ED LOS locally (54).

Considering that the outcome in this analysis was the overall LOS, further analyses of the more detailed KPIs collected through the Ada’a program, namely, door to doctor, doctor to decision, and decision to disposition, in addition to this overall KPI, will provide the necessary information to develop tools/policies for improvement. We also recommend measuring ED LOS at the cluster level of the business units, as proposed by the new model of care introduced in the healthcare transformation plan of Vision 2030, and at the regional level of the 13 administrative regions of Saudi Arabia (55, 56). With highly varying socioeconomic levels among regions, we expected to find regional differences due to these variations. A closer examination of the method of data entry owing to the stark differences observed here is also necessary (57).

### Strengths and limitations

This study is the first to use national data of this magnitude to assess the performance of EDs in terms of their overall LOS. The extremely large sample size allowed for the identification of clear LOS patterns and descriptions. However, this study does not claim a direct causal relationship between ED LOS and any of the variables, owing to the nature of its design. More importantly, this study was not able to assess whether other factors such as resources, crowdedness, hospital throughput, and patient flow have influenced ED LOS since they were not collected within the Ministry of Health dataset. Additionally, comparisons across the years, specifically for 2019, were limited because data collection commenced in September 2019. Despite these shortcomings, this study remains a valuable resource for future planning and policymaking.

### Future research

As this study bridged the pre-COVID-19 through the COVID-19 pandemic periods, the effect of the COVID-19 pandemic on ED LOS should be studied in the future.

## Conclusion

This study reports ED visit LOS throughout Saudi Arabia along with related factors. The findings showed that LOS showed statistically significant differences in relation to many variables. We offer this study as a scientific basis for future planning and policymaking to improve the quality of care in the Saudi healthcare system. We have also provided suggestions for further research based on these findings.

## Data availability statement

The raw data supporting the conclusions of this article are available from the Ministry of Health upon reasonable request to the corresponding author.

## Ethics statement

The studies involving humans were approved by the MOH and Imam Abdulrahman Bin Faisal University institutional review boards. The studies were conducted in accordance with the local legislation and institutional requirements. The participants provided their written informed consent to participate in this study.

## Author contributions

AbA: Conceptualization, Data curation, Formal analysis, Investigation, Methodology, Project administration, Resources, Software, Supervision, Validation, Visualization, Writing – original draft, Writing – review & editing. MM: Conceptualization, Data curation, Formal analysis, Investigation, Methodology, Project administration, Resources, Software, Validation, Visualization, Writing – original draft, Writing – review & editing. RA: Conceptualization, Data curation, Formal analysis, Funding acquisition, Methodology, Project administration, Resources, Software, Validation, Visualization, Writing – original draft, Writing – review & editing. AhA: Conceptualization, Data curation, Formal analysis, Investigation, Methodology, Project administration, Resources, Software, Validation, Visualization, Writing – original draft, Writing – review & editing. MAM: Conceptualization, Data curation, Formal analysis, Investigation, Methodology, Project administration, Resources, Software, Supervision, Validation, Visualization, Writing – original draft, Writing – review & editing. AYA: Conceptualization, Data curation, Formal analysis, Investigation, Methodology, Project administration, Resources, Software, Supervision, Validation, Visualization, Writing – original draft, Writing – review & editing. RSA: Conceptualization, Data curation, Formal analysis, Investigation, Methodology, Project administration, Resources, Software, Supervision, Validation, Visualization, Writing – original draft, Writing – review & editing. MA: Conceptualization, Data curation, Formal analysis, Investigation, Methodology, Project administration, Resources, Software, Supervision, Validation, Visualization, Writing – original draft, Writing – review & editing.
